# Nanoparticles for Targeted Delivery of Active Agents against Tumor Cells

**DOI:** 10.1155/2012/528123

**Published:** 2012-07-05

**Authors:** Rassoul Dinarvand, Paulo Cesar de Morais, Antony D'Emanuele

**Affiliations:** ^1^Nanotechnology Research Centre, Faculty of Pharmacy, Tehran University of Medical Sciences, Tehran 1417614411, Iran; ^2^Núcleo de Física Aplicada, Instituto de Física, Universidade de Brasília, 70910-900 Brasília, DF, Brazil; ^3^School of Pharmacy and Pharmaceutical Sciences, University of Central Lancashire, Preston PR1 2HE, UK

Cancer therapy may still be considered as one of the most challenging issues in pharmaceutical sciences. Various new technologies and molecules have been developed to meet the needs of this devastating disease, but there is a long way to go. One of the most important challenges is finding a drug molecule or drug delivery system which only influences the cancer cells. This has not been achieved so far even though a great deal of research has focused on this goal. Most of the cytotoxic agents used for cancer treatment are toxic in nature and not only have an effect on tumor cells, but also normal cells are damaged when exposed with them. As a result, patients may experience intensive side effects which may lead to an early termination in chemotherapy due to their severity.

Targeting the cancer cells specifically and selectively seems to be the best solution for this obstacle. Targeting the cancer cells occurs via two different strategies: passive targeting and active targeting. The conventional approach used for cancer therapy may be considered a sort of passive targeting. Cytotoxic agents used for cancer treatment select the cancer cells due to their faster growth rate. Inevitably, normal fast growing cells and normal fast dividing cells may damage following conventional chemotherapy. In 1986, Matsumura and Maeda brought up a new concept in passive targeting called enhanced permeation and retention (EPR) effect which was widely used in many researches as an approach for targeting tumor cells. In this strategy, the drug delivery system benefits from defective vasculature, fenestrations, and poor lymphatic drainage in tumor tissues. Utilizing molecular targeting moieties is another strategy which is widely used in new researches for targeting tumor cells actively and diminishing chemotherapy side effects.

This special issue will primarily address new approaches for passive and active targeted cancer therapy with a focus on nanoparticle formulations specially those ones that specifically and selectively target tumors.

There is a peper which describes using a natural protein, atelocollagen for oligonucleotide delivery, and evaluating its biological function. The researchers found that when oligonucleotides were used concomitantly with atelocollagen, paracellular flux was enhanced, but when each of these two constituents of the formulation was used separately, no effect was observed. Another paper reviews poly(lactic-co-glycolic acid)- (PLGA-) based nanoparticles especially echogenic ones to enhance gene delivery. Ultrasound may be used to enhance localized gene delivery in tumors. On the other hand, nanoparticles themselves may utilize targeting mechanisms to enhance the targeting potential of the current system. Another paper describes microemulsions in the form of nanocarriers for passive tumor targeting. In this study, an experimental design layout was used to optimize the excipients selection for microemulsion nanocarriers. These nanocarriers possess the ability of solubilizing hydrophobic drugs and physical stability alongside with their capability of passive targeting. In another paper human serum albumin was used as a naturally occurring protein to create nanoparticles for enhanced drug delivery in breast cancer. Albumin-based nanoparticles were further cationized by polyethylenimine. It was shown that delivery efficacy was improved by passive EPR strategy.

One of the papers also reviews carbon nanotubes for cancer therapy and designing drug delivery systems. Carbon nanotubes present a great canvas for surface engineering and creating novel targeted nanoparticles. Another paper presents glucan particles which actively target cancer cells. Glucan particles which benefit from *β*-glucan derived from the cell wall of *Saccharomyces cerevisiae* provide receptor-mediated uptake by phagocytic macrophage cells who express *β*-glucan receptors. Different types of nanoparticles were incorporated or surface bounded to these glucan particles to deliver doxorubicin to macrophages. Macrophages further migrate into solid tumors carrying the cytotoxic agent to the target tissues like a Trojan horse, while they are resistant to the effects of doxorubicin due to their nondividing differentiated nature.

Another paper reviews noble metal nanoparticles with a wide variety of biomedical applications which are used for both therapeutic and diagnostic purposes. They also present facile surface chemistry and the possibility of functionalizing on their surfaces.

Finally, in one of the papers, a lipoplatin formulation consisting of liposomal encapsulated cisplatin is reviewed. The purpose of this formulation is to become a substitute for the original cisplatin and has gained positive results in clinical trials. Lipoplatin gives 200-fold higher concentrations in tumor tissues in comparison with cisplatin once more because of the compromised endothelium of tumor tissues.

To sum up, this special issue tries to provide the readers some insight about the targeted nanoparticles which are designed for cancer therapy both for small molecular drugs and gene delivery systems. 

*Rassoul Dinarvand* *Rassoul Dinarvand* *Paulo Cesar de Morais* *Paulo Cesar de Morais* *Antony D'Emanuele* *Antony D'Emanuele*

